# Developing a Sufficient Protocol for the Enhancement of α-Glucosidase Inhibitory Activity by *Urena lobata* L. Aeroponic Hairy Roots Using Exogenous Factors, a Precursor, and an Elicitor

**DOI:** 10.3390/plants9040548

**Published:** 2020-04-23

**Authors:** Dai Minh Cao, Phuong Thi Bach Vu, Minh Thi Thanh Hoang, Anh Lan Bui, Phuong Ngo Diem Quach

**Affiliations:** 1 Laboratory of Plant Biotechnology, Department of Plant Biotechnology and Biotransformation, University of Sciences, Ho Chi Minh City 7000, Vietnam; cmdai@hcmus.edu.vn (D.M.C.); vtbphuong@hcmus.edu.vn (P.T.B.V.); httminh@hcmus.edu.vn (M.T.T.H.); blanh@hcmus.edu.vn (A.L.B.); 2 Vietnam National University, Ho Chi Minh City 7000, Vietnam

**Keywords:** *Urena lobata*, hairy root, aeroponics, phenylalanine, chitosan, synergistic effect

## Abstract

Aeroponics is considered as a potential method for the culture of herbal plants due to the high growth rate, quantity and quality enhancement of secondary metabolites, and substantial environmental progress associated with this method. The aim of this study was to develop a sufficient protocol for successful *Urena lobata* hairy root induction by *Agrobacterium rhizogenes* ATCC 15834, using a precursor and elicitor to enhance α-glucosidase inhibitory activity (GIA) of aeroponic hairy roots (AHRs) in greenhouse conditions. In this study, we found that the optimized procedure (10 min, Woody plant medium (WPM), 1/25 salt strength) had an outstanding effect with a reduction in the rooting time (RT), promotion of the rooting rate (RR), and increase in the fresh weight (FW) and dry weight (DW) compared with the original procedure (30 min, Murashige and Skoog (MS) medium, 1/25 salt strength) after 30 days of culture. The highest DW, GIA, flavonoid (FLA) and phenolic (PHEL) contents were observed for individual addition of 10 mM phenylalanine (PA) or 50 mM chitosan (CS) in the late exponential phase (eighth week) with 15 days of elicitation compared to the control AHRs. However, individual treatment was less effective than the combination of the two. Positive correlations among the GIA, FLA and PHEL indicate that AHRs accumulated phenolic compounds, leading to an increase in the GIA by a synergistic effect. In conclusion, the culture of *Urena lobata* AHRs with PA and CS is an efficient procedure to produce GIA material in greenhouse conditions.

## 1. Introduction

*Urena lobata* originates from the wilderness of North and South America and Asian countries such as Indonesia, Philippines and Vietnam. Traditionally, leaves and roots have been used to treat and diagnose colic, malaria, fever, wounds, and toothache [1]. In a previous study, *Urena lobata* root extract was demonstrated to possess antioxidative activity by strongly inhibiting lipid peroxidation and scavenging hydroxyl and superoxide radicals in vitro [2]. In addition, in Nigeria, *Urena lobata* is known as an antidiabetic herbal that can provide long-term control of hypoglycemia in normal rabbits [3]. In 2010, a study of aqueous extracts of *Urena lobata* in streptozotocin-induced diabetic rats revealed that the root extract of *Urena lobata* is more effective than the leaf extract at handling the raised blood glucose levels of diabetic rats [4].

The treatment of type II diabetes is sophisticated in terms of the variety of risk factors, one of which is the considerably rapid development of postprandial hyperglycemia [5]. Postprandial hyperglycemia occurs through the reaction of α-glucosidase and α-amylase in order to produce glucose. Therefore, by restricting or reducing the activity of these enzymes, the occurrence of postprandial hyperglycemia should decrease. The inhibition of α-glucosidase activity leads to a deterioration in disaccharide hydrolysis which has substantial long-term effects on glycemic index control in diabetic patients [6]. In recent years, natural medicines from plants have been used as useful treatments without any side effects.

Hairy roots (HRs) are established from the interaction between *Agrobacterium rhizogenes* and the host plant (gene transformation from bacteria to the genome of the plant) [7]. There are numerous merits that accrue from the collaboration; in particular, a high growth rate without the dependence of any exogenous hormones, genetic stability, and a high branching rate. In addition, the ability to produce secondary metabolites remains stable. Hence, the HR cultural technique is considered to be an attractive substitute for the manufacture of tremendously valuable natural secondary metabolites instead of bud or callus cultures [8].

Accompanied by the significant progress of modern techniques, aeroponics is a new method that has been adapted from classical hydroponics. Compared with hydroponics, aeroponics has more considerable benefits such as the provision of water and mineral nutrients, a rapid increase in growth rate, improved quantity and quality of secondary metabolites, and substantial environmental progress [9].

Feeding precursors and elicitors into the plant biosynthesis pathway is considered an effective approach for the production of secondary metabolites. L-phenylalanine (PA) is an aromatic amino acid and a substrate of phenylalanine ammonia lyase (EC 4.3.1.5). This enzyme catalyzes the transformation of PA into trans-cinnamic acid and is the first step of the biosynthesis pathway of plant phenolic compounds [10]. Chitosan (CS), a polycationic polymer of β-1,4 linked D-glucosamine, is a bioactive agent known to be an efficient elicitor to build up the production of plant secondary metabolites. By eliciting plant defense protection, CS remarkably enhances this production of phenolic compounds through signal transduction and phytoalexin production [11]. In addition, the secondary metabolites produced from the elicitation of CS were studied and found to have a strong correlation to the reaction of PA and phenylalanine ammonia lyase [12].

In our early research, we successfully cultured the HRs of *Urena lobata* in vitro and in an aeroponics system. We also found that the ethanol extract obtained from aeroponic hairy roots (AHRs) has stronger α-glucosidase inhibitory activity (GIA) than in vitro HRs (GIA = 0.15 (μg/mL)^−1^). Moreover, it was shown to have the same value (0.21 (μg/mL)^−1^) as natural root (0.21 (μg/mL) ^−1^) [13]. We also evaluated the activity of *Urena lobata* hairy root in alloxan-induced diabetic mice. The results revealed that the blood glucose level in the group treated with one of the three extracts showed a significant decrease as compared with the untreated group. However, this research showed that the hypoglycemic ability of AHRs was still slightly lower than that of the natural roots, possibly due to the culture duration being insufficient for the accumulation of secondary metabolites in AHRs [14].

The aims of this work were to develop a sufficient procedure for successful *Urena lobata* AHR induction by *Agrobacterium rhizogenes* ATCC 15834 and to enhance the GIA of AHRs by using it alone or in combination with PA (precursor) or CS (elicitor).

## 2. Results

### 2.1. Optimization of Exogenous Factors Optimizing for AHRs and rol Gene Detection

**Figure 1:**
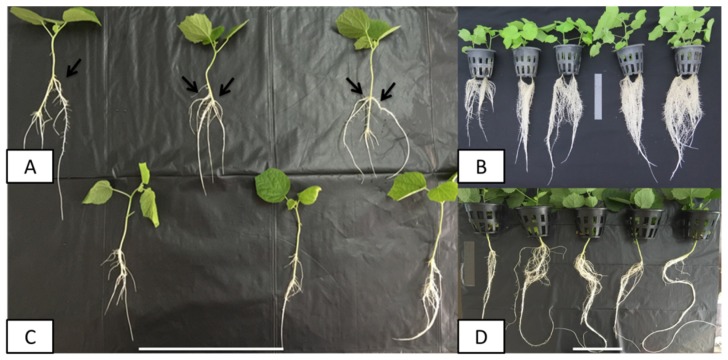
AHR induction after 7 days. (**A**,**C**) Transformed and non-transformed roots after 7 days; (**B**,**D**) transformed and non-transformed roots after 30 days. The black arrows indicate HR formation. Scale bar = 20 cm.

Hairy root induction and root growth is affected by different factors such as plant age, explants, *Agrobacterium* strains, infection time, media composition, and co-inoculation time [15]. In our previous study, we established a protocol for AHRs cultures of *Urena lobata* [13]. To optimize this protocol and enhance the GIA, we investigated the effects of infection time, media composition, and the ratio of mineral salt strength on the rooting time (RT), rooting rate (RR), fresh weight (FW) and dry weight (DW) (Figure 1 and Table 1). The plantlets were infected with the *Agrobacterium rhizogenes* ATCC 15834 suspension at different time points. An infection time of 10 min was optimal in terms of transformation efficiency and root growth, compared with 30 min or 60 min. The root initials were observed at the infection sites as early as three days after inoculation. Moreover, the root biomass produced by 10 min of infection was higher after 30 days compared to that produced by 30 min or 60 min of infection. Therefore, an infection time of 10 min was selected for the optimized procedure.

The media composition is another important factor for hairy root induction and growth, as well as for the production of secondary metabolites [16,17]. Therefore, we compared the effects of different induction media such as Murashige and Skoog (MS) [18], Gamborg B5 (B5) [19] and Woody plant medium (WPM) [20] on the RT. Our results showed that WPM enhanced the root initials by reducing the RT, and it also induced the highest RR. Then, we compared the different mineral ratio strengths in WPM. Roots grown on WPM 1/25 showed the highest biomass production.

To examine whether the combination of these improved factors could increase the transformation efficiency and root growth, we generated an optimized procedure with the following parameters: infection time of 10 min, WPM, and 1/25 salt strength for the growth medium. Compared with the original procedure, the optimized procedure resulted in improvements in RT and RR. In fact, the FW and DW of the AHRs grown with the optimized procedure increased by 1.9-fold and 3.0-fold, respectively, compared with the original procedure (Table 2).

**Figure 2:**
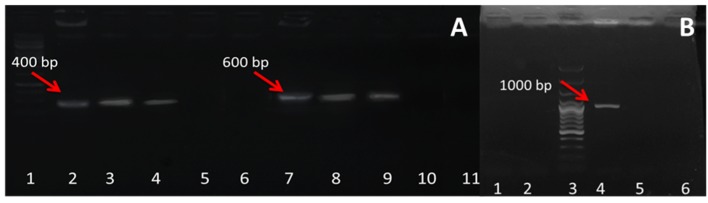
PCR detection of *rolB*, *rolC*, and *virG*. (**A**) PCR amplification of 400 bp fragments of *rolB* and 600 bp fragments of *rolC*. The detection of *rolB*: lane 1 = ladder 100 bp; lane 2 = positive control; lanes 3 and 4 = AHRs; lanes 5 and 6 = negative control of aeroponic roots. Detection of *rolC*: lane 7 = positive control; lanes 8 and 9 = AHRs; lanes 10 and 11 = negative control of aeroponic roots. (**B**) The absence of *virG* in AHRs: lanes 1 and 2 = negative control of aeroponic roots; lane 3 = ladder 1000 bp; lane 4 = positive control; lanes 5 and 6 = AHRs.

The integration of Ri T-DNA into the genomes of plant cells caused the formation of AHRs, in which *rol* genes were harbored after seven days (Figure 2). After infection with *Agrobacterium rhizogenes* ATCC 15834, the genetic status of the established roots was assessed using a polymerase chain reaction (PCR)-based analysis of *rol*B and *rol*C genes. In addition, PCR analysis using gene-specific primers for *vir*G was applied to exclude bacterial contamination in the culture. *Agrobacterium rhizogenes* (colony PCR) served as the positive control, and DNA from the non-transformed seedling roots served as the negative control. Our results showed PCR products at 423 bp and 625 bp for the *rol*B and *rol*C fragments, respectively, in the positive control and hairy root samples, which indicated successful genetic transformation (Figure 2A). In contrast, no product of PCR amplification was detected for the *vir*G gene in the same hairy root lines, indicating the absence of *Agrobacterium rhizogenes* (Figure 2B).

**Figure 3:**
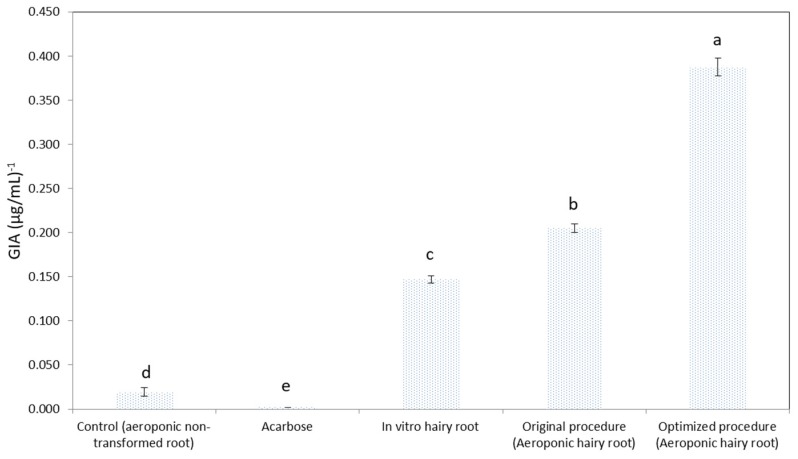
Evaluation of *Urena lobata* AHRs α-glucosidase inhibitory activity (GIA). Error bars represent standard deviations. The values in a line marked with different lower cases letters denote significant differences between samples at *p* < 0.05 (Duncan’s multiple range test).

We evaluated the GIA from in vitro hairy roots, aeroponic non-transformed roots, AHRs from the original procedure, and AHRs from the optimized procedure. The AHRs had 2.6-fold and 19.8-fold higher GIA than in vitro hairy roots and aeroponic non-transformed roots, respectively. The GIA from AHRs produced by the optimized procedure increased by 1.9-fold in comparison to the AHRs cultured in the original procedure (Figure 3). Collectively, the HRs of *Urena lobata* could be generated and cultured in an aeroponics system to obtain a high yield of valuable material resource.

### 2.2. Kinetics of Growth, α-Glucosidase Inhibitory Activity, and Phenolic and Flavonoid Contents of AHRs

**Figure 4:**
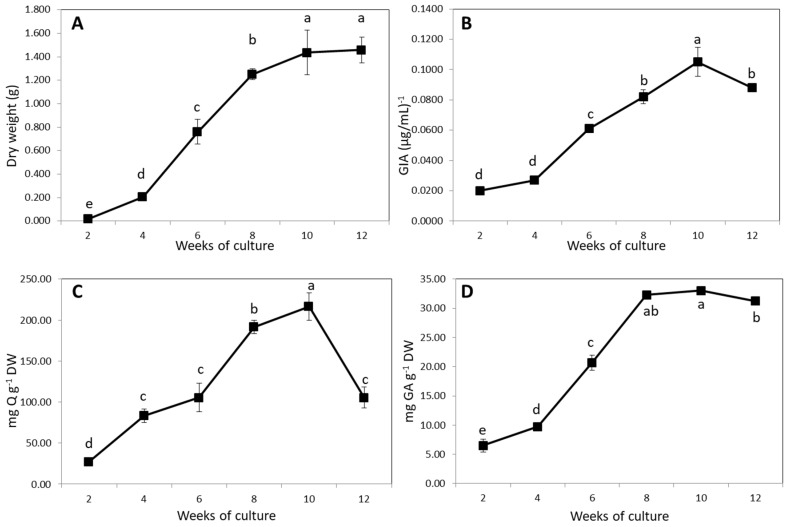
Kinetics of growth. (**A**), GIA (**B**), PHEL (**C**) and FLA (**D**) of *Urena lobata* AHRs cultured for 12 weeks. Each value in the curve represents the average of three independent cultures; error bars represent standard deviations. The values in a line marked with different lower-case letters denote significant differences between samples at *p* < 0.05 (Duncan’s multiple range test).

After induction with *Agrobacterium rhizogenes* ATCC 15834 in the optimized procedure, the *Urena lobata* plantlets were cultured with aeroponics for 12 weeks. As shown in Table 3 and Figure 4, there was a clear positive correlation among DW, GIA, and the flavonoid (FLA) and phenolic (PHEL) contents. There was a significant increase from weeks 4 to 10, and then values were maintained or markedly decreased until the 12th week. Maximum GIA, FLA, and PHEL values of 1.44 g, 0.11 (µg/mL)^−1^, 216.67 mg Q g^−1^ DW and 33.03 mg GA g^−1^ DW, respectively, were observed for AHRs at the 10th week of culture.

### 2.3. Effect of Phenylalanine Feeding on Growth, α-Glucosidase Inhibitory Activity, Total Flavonoids and Phenolics Contents of AHRs

**Figure 5:**
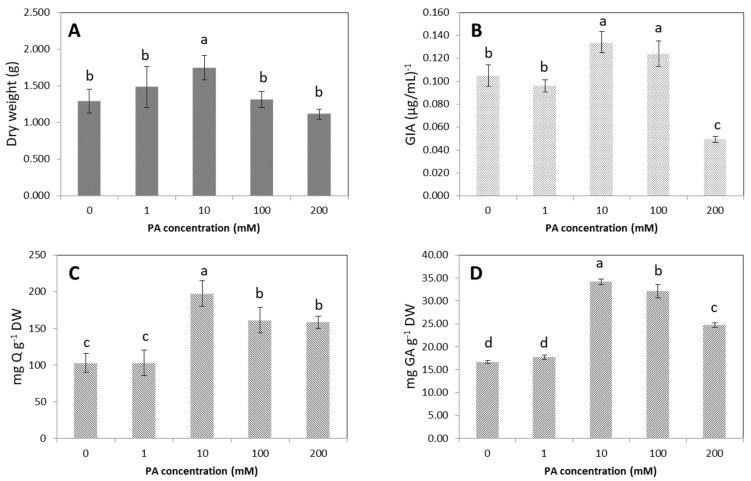
The effects of various phenylalanine (PA) concentrations on AHRs. (**A**) DW, (**B**) GIA, (**C**) FLA (**D**) PHEL. Error bars represent standard deviations. The values in one line marked with different lower-case letters denote significant differences between samples at *p* < 0.05 (Duncan’s multiple range test).

At the 10th week of culture, AHRs were collected and the DW, GIA, FLA and PHEL were evaluated after adding various PA concentrations. As shown in Figure 5, by feeding 10 mM PA, significant elevations in the DW (1.4-fold), GIA (1.3-fold), FLA (1.9-fold), and PHEL (2.1-fold) of AHRs occurred in comparison with the control. After adding PA at higher concentrations (100 mM and 200 mM), the AHRs showed lower GIA and lower production of secondary metabolites compared with those where 10 mM PA was added. For example, the AHRs cultured in the medium with 200 mM PA added showed dramatic decreases in GIA (2.7-fold) and PHEL (1.4-fold).

Pearson’s correlation matrix (Table 3) demonstrated that DW showed a significant positive correlation with FLA. FLA exhibited a significant positive correlation with PHEL, but there was no positive association between GIA and the accumulation of phenolic compounds in the treatment AHRs (FLA and PHEL).

### 2.4. Effect of Chitosan Dosages and Exposure Time on Growth, α-Glucosidase Inhibitory Activity, and Total Flavonoid and Phenolic Contents of AHRs

**Figure 6:**
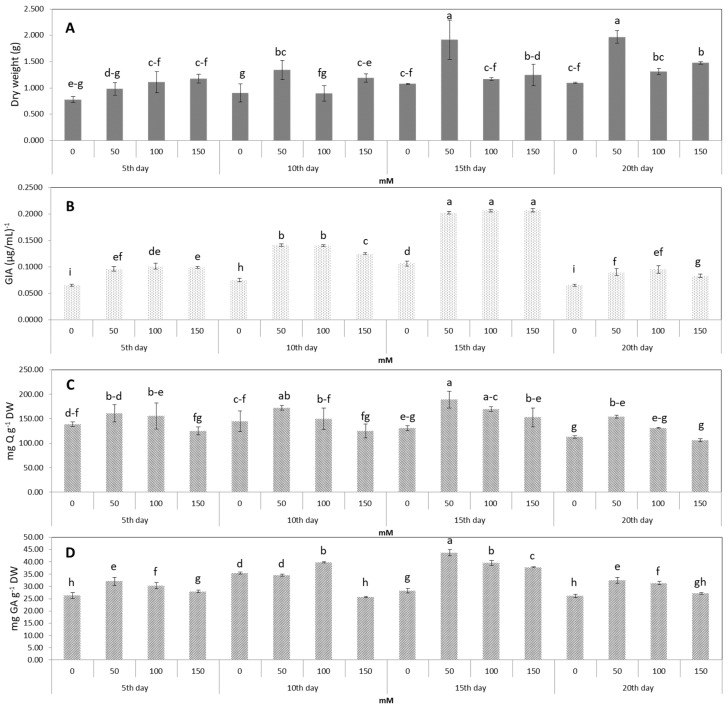
Effect of the CTS and exposure time (ET) on AHRs. (**A**) DW, (**B**) GIA, (**C**) FLA (**D**) PHEL. Error bars represent standard deviations. The values in one line marked with different lower-case letters denote significant differences between samples at *p* < 0.05 (Duncan’s multiple range test).

After the elicitation of different chitosan dosages (CTS), apart from the treatment with 50 mM CS, the other concentrations showed no significant difference in biomass accumulation. With the addition of 50 mM CS, the DW of AHRs significantly increased until the 15th day of elicitation, with growth ending on the 20th day (Figure 6A). Regarding other parameters, CS feeding with different CTS and exposure time (ET) showed obvious improvements in GIA and the production of secondary metabolites by the elicited AHRs. Among these, the addition of 50 mM CS after 15 days of elicitation had an exceptional effect. It led to the most significant improvements in the controls: a 2.0-fold increase in GIA, 1.4-fold increase in FLA, and 1.5-fold increase in PHEL (Figure 6B–D). On the 20th day of elicitation, all the CTS showed large decreases in GIA, FLA and PHEL.

As shown in the Pearson’s correlation matrix (Table 3) of the CS treatment, the GIA was found to correlate positively with both FLA and PHEL. In contrast to phenylalanine treatment, the DW not only showed no significant correlation with the FLA, but also with the GIA and PHEL.

### 2.5. Combined Effect of Phenylalanine and Chitosan on Biomass and α-Glucosidase Inhibitory Activity

**Figure 7:**
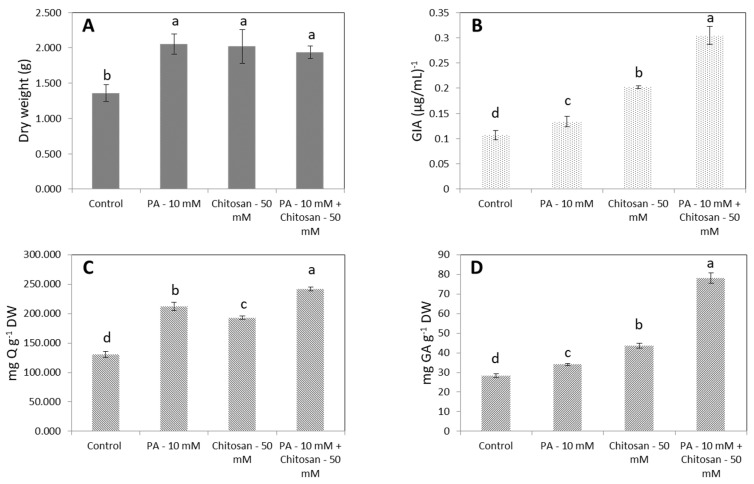
The effects of the combination of PA and chitosan (CS) on AHRs. (**A**) DW, (**B**) GIA, (**C**) FLA (**D**) PHEL. Error bars represent standard deviations. The values in a line marked with different lower-case letters denote significant differences between samples at *p* < 0.05 (Duncan’s multiple range test).

To evaluate the combined effect of PA and CS, *Urena lobata* plantlets were cultured in the optimized medium with the addition of 10 mM PA. Subsequently, at the eighth week, 50 mM chitosan was implemented, and the plants were finally harvested after 15 days. As shown in Figure 7, the combination of 10 mM PA with 50 mM CS had an outstanding effect after 15 days of elicitation. The DW increased by 1.4-fold, the GIA increased by 3.0-fold, the FLA increased by 1.9-fold and the PHEL increased by 2.8-fold compared with the control. In contrast, individual feeding of PA or CS was not associated with significant increases in GIA or the production of secondary metabolites. For instance, PA could only increase the GIA by 1.3-fold, the FLA by 1.6-fold, and the PHEL by 1.2-fold and CS could only promote the GIA by 1.9-fold, the FLA by 1.5-fold and the PHEL by 1.5-fold compared with the control.

## 3. Discussion

### 3.1. Optimization of Exogenous Factors for the AHRs and rol Gene Detection

According to Thwe et al., developing an efficient procedure for successful HR induction by *Agrobacterium rhizogenes* is the key step in determining a culturing method for the mass production of secondary metabolites [21]. In this study, the infection time, type of medium and ratio of mineral salt strength were considered in the development of a procedure to enhance the rooting efficiency and growth of *Urena lobata* AHRs. Generally, a short infection time (15–30 min) was not effective, probably due to the insufficient time for bacterial infection [22]. However, the highest transformation rate was achieved with only a 10 min infection time, and not with 30 min or 60 min infection times. This result may be because *Urena lobata* is quite vulnerable to the *Agrobacterium rhizogenes* ATCC 15834 strain. Similarly, Phuong et al. found that *H. sabdariffa* L. (also belonging to the Malvaceae family) showed a high transformation rate with only 20 min of infection time [23]. On the other hand, medium composition and mineral salt strength ratio usually have indirect effects but are sufficient for the establishment of HRs. Appropriate modification of the medium composition and mineral salt strength ratio would help to increase the biomass accumulation of AHRs. Specifically, this modification changes the NO_3_^−^/NH_4_^+^ ratio, leading to significant differences with each treatment. According to Sharafi et al., an increase in the NO_3_^−^/NH_4_^+^ ratio up 3-fold will reduce the biomass production of *Papaver bracteatum* HRs [24]. The NO_3_^−^/NH_4_^+^ ratio of B5 medium is about 3-fold higher than WPM, resulting in a reduction in biomass [25]. In addition, it is necessary to determine a species-specific medium for the accumulation of biomass, as the ratio of mineral salt strength requirement for growth differ among species. The amount by which the mineral salt strength is over the essential level may inhibit growth of AHRs due to osmotic stress. Low-mineral salt strength may decrease the biomass accumulation of AHRs because nutrients are quickly depleted [26].

AHRs established after the induction of *Agrobacterium rhizogenes* have a higher growth rate and more accumulation of secondary metabolites than the aeroponic non-transformed roots. Moreover, when AHRs were cultured in the optimized procedure, it revealed an outstanding effect in comparison with the original procedure. Many previous studies have demonstrated that the integration of *rol* genes indirectly affected the expression of enzymes belonging to the biosynthesis pathway of secondary metabolites, leading to an increase in the bioactivity level [27,28]. Tusevski et al. found that the integration of *rol*B into plant cells through the *Agrobacterium rhizogenes* transformation process activated the phenylalanine ammonia lyase activity of *H. perforatum*, leading to increased flavonoid production [29]. Meanwhile, HRs grown by aeroponics not only inherited the integration of *rol* genes but were also cultured in a medium under controlled nutrients. Moreover, the supported carbohydrates from photosynthesis of the aerial part probably led to the higher GIA and greater production of secondary metabolites.

### 3.2. Kinetics of Growth, α-Glucosidase Inhibitory Activity, and Phenolic and Flavonoid Contents of Hairy Root of Urena lobata

In our previous study, AHRs cultured for only 30 days reached a level of GIA equal to natural roots [14]. Moreover, many studies have already reported that HRs accumulate phenolic compounds in amounts that lead to a significant increase in bioactivity that is comparable to, or even greater than, that present in intact plants or normal root cultures [30,31]. In fact, some flavonoids with potential bioactivity, such as quercetin and kaempferol, are found in *Urena lobata* [32,33]. In order to determine the appropriate time of application and types of precursor and elicitor that are appropriate for the enhancement of inhibitory activity, the duration of AHR culture was extended to up to 12 weeks, accompanied by the evaluation of DW, GIA, FLA, and PHEL. As shown in Figure 4 and Table 2, GIA showed a strong positive correlation with the total FLA and PHEL contents. Therefore, PA and CS were applied in this farming strategy. Regarding the feeding time, PA was added on the first day of culture, and the culture was harvested after 10 weeks because the GIA of the AHRs declined in the 12th week. Meanwhile, week eight was determined to be a suitable stage for adding CS, as this was when the AHRs achieved the highest DW and began to accumulate secondary metabolites.

### 3.3. Effect of Phenylalanine Feeding on Growth, α-Glucosidase Inhibitory Activity, and Total Flavonoid and Phenolic Contents of Urena lobata Aeroponic Hairy Root

**Figure 8:**
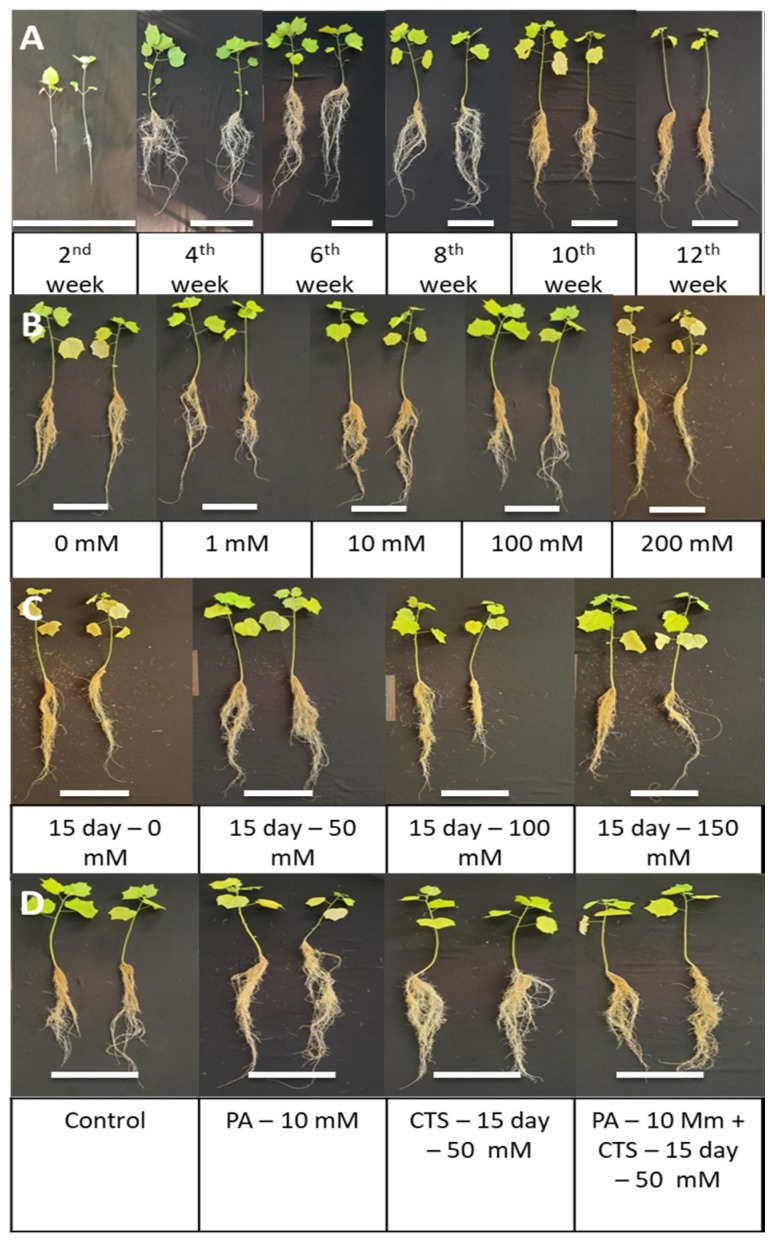
AHR phenotypes were cultured and affected by various treatments with PA and CS. (**A**) AHRs culture at 12 weeks. (**B**) Comparison of growth with various PA concentrations. (**C**) Comparison of growth with various CTS and ET. (**D**) Comparison of individual and synergistic effects of growth. Scale bar = 20 cm.

In our research, AHRs increased biomass production when PA was applied at the appropriate dosage (10 mM), which showed that PA plays a role as an additional amino acid (organic nitrogen) in the first stage of culture. When the AHRs accumulated adequate biomass, PA increased the FLA and PHEL by playing a role as an initial substrate of the phenolic biosynthesis pathway. In this plant pathway, one of the first and key steps is the deamination of PA into trans-cinnamic acid (carbon skeletons of the phenylpropanoid pathway) and ammonia, a reaction catalyzed by phenylalanine ammonia lyase. These carbon skeletons may continuously participate in the shikimate pathway to produce phenolic compounds in the AHRs of *Urena lobata*, resulting in significant increases in the production of flavonoids and phenolics. In contrast, when a high concentration of PA (200 mM) was added, there were lower levels of GIA accompanied by bioactive compounds than in other treatments. In this condition, the color of AHRs turned brown (Figure 8). This result indicates that the presence of a high concentration of precursors may induce stress in plant cells, resulting in cell destruction. Shinde et al. showed that the use of excessive PA had a negative effect on the isoflavone content of *P. corylifolia* HRs [34]. Therefore, having an appropriate concentration of the precursor is a fundamental factor. In addition, feedback inhibition to the metabolic pathway due to excess precursor must be considered during product optimization [35]. In our previous experiment in an in vitro HR culture of *Urena lobata*, PA feeding at a high level (100 mM and 200 mM) did not show any significant differences in biomass accumulation in comparison with low levels (1 mM and 10 mM) or the control HRs. However, the GIA decreased greatly (by 3.8-fold) at the concentration of 100 mM compared with the control (data not shown). Accordingly, aeroponics culture of *Urena lobata* at an appropriately low concentration is an interesting technique that can be used to enhance the productivity of secondary metabolite accumulation, leading to an increase in the GIA of AHRs.

### 3.4. Effect of Chitosan Dosages and Exposure Time on Growth, α-Glucosidase Inhibitory Activity, and the Total Flavonoid and Phenolic Contents of Urena Aeroponic Lobata Hairy Root

The farming strategy of secondary metabolites in plant cells is usually divided into two phases: accumulation of biomass in the first phase and an increase in the concentration of secondary metabolites in the second phase [25]. The growth rate of *Urena lobata* AHRs at the eighth week of culture was the highest, so that allowed us to step into the second phase with CS addition. The establishment and growth of HRs accompanied by plantlets show superior capacity to in vitro HRs. In this study, the *Urena lobata* plantlets induced by *Agrobacterium rhizogenes* had sufficient ability to respond to the elicitor at the appropriate concentration, which led to an increase in GIA as well as the accumulation of FLA and PHEL, but not a decline in biomass by the time of elicitation. In addition, CS was found to be an elicitor with less detrimental effects on the growth of explants. For example, marked increases in the cell biomass of *Ocimum basilicum* L., *Ocimum sanctum* L. and *Ocimum gratissimum* L. were observed when CS was added, demonstrating that it could effectively enhance cell biomass in a shorter amount of time, and hence it can be used for effective induction of secondary plant metabolites [36]. Another result from Yanjie et al. revealed that CS also plays a role in increasing the nitrogen and phosphor consumption of *Podocarpus macrophyllus* and *Taxus cuspidata* tree seedlings, resulting in remarkable promotion of biomass accumulation [37]. The results implied that CS is an effective elicitor that can enhance the GIA by increasing the production of the flavonoid and phenolic contents in AHR cultures of *Urena lobata*. However, at the longest ET (20th day), the GIA, FLA and PHEL decreased dramatically and the DW only remained at the level of the 15th day, possibly due to the depletion of nutrients in the medium. When both primary and secondary metabolic pathways were activated at the same time, the plantlets consumed nutrients at a higher speed than normal, resulting in a deficiency in nutrients by the 20th day. It is possible that 15 days of treatment was too short to enable the accumulation of the tested stimulators in an amount that was growth-suppressing or toxic to root cells but was long enough to stimulate different metabolic points of FLA and PHEL biosynthesis. In addition, extending the ET may lead to the diffusion of natural endogenous compounds into the environment and cause detrimental effects on the AHRs. Similar to Shinde et al., the daidzein content in the HRs of *P. corylifolia* decreased by 1.5-fold when the time of elicitation was prolonged by up to two-fold and was accompanied by a reduction in biomass [34]. A previous study showed that CS may induce a biosynthesis pathway to produce secondary metabolites originating from PA through cinnamic and coumaric acids [12]. Similar to Udomsuk et al., CS increased the accumulation of total isoflavonoids in the HRs of *Pueraria candollei* by up to 2.8 times [38]. CS is one of the components in the cell wall of Zygomycetes fungi. It may accelerate the concentration of phenylalanine ammonia lyase by activating the protection of plant cells [39]. To respond to pathogenic factors, plant cells firstly produced chitosanase, which breaks down chitosan (polymer form) into shorter chains (chitosan oligomer form). These chitosan oligomers have been shown to play a fundamental role in inducing the level of phenylalanine ammonia lyase production [40]. The concentration of this enzyme in soybean leaves was increased by up to approximately two-fold after 36 h of treatment with CS, which may lead to the induction of other secondary metabolites produced by the phenylpropanoid pathway [41]. For that reason, the elicited mechanism of CS is related to PA consumption and leads to a significant increase in the concentration of natural compounds. Therefore, here, we investigated whether the combination of PA with CS would have a positive effect by promoting the GIA.

### 3.5. Combined Effect of Phenylalanine and Chitosan on Biomass and α-Glucosidase Inhibitory Activity

With the combined feeding of PA and CS at appropriate concentrations and time point, AHRs may accumulate higher amounts of secondary metabolites from the phenylpropanoid pathway, leading to an increase in the GIA. In the individual PA treatment, the bivariate correlation showed that although there were positive correlations with FLA and PHEL, GIA was not promoted (Table 3). This issue may be because PA is not a specific precursor to increase the FLA and PHEL possessed by the GIA in AHRs. The products of phenylalanine ammonia lyase and PA will continuously enter the phenylpropanoid pathway to make various flavonoid and phenolic compounds. Meanwhile, in the individual treatment of CS, GIA exhibited a significant positive relation with FLA and PHEL (Table 3). CS elicited not only the PAL, but also other secondary metabolic enzymes in the phenylpropanoid pathway. These enzymes may relate to the production of specific phenolic compounds that lead to the elevation of the GIA in AHRs. As shown in Figure 7C,D, the FLA after treatment with CS alone was lower, but the GIA and PHEL were higher than following PA treatment. Similarly to what was shown by Funk and Brodelius, CS treatment led to significant increases in both phenylalanine ammonia lyase and 4-hydroxycinnamate: coenzyme A ligase activity in suspension cultures of *Vanilla planifolia*. CS promoted the production of a significantly higher concentration of two intracellular phenolic compounds (4-coumaric and sinapic acid) than in the control [42]. Flocco and Giulietti observed the peroxidase activity by treatment of HRs of *Armoracia lapathifolia* with CS [43]. The activity increased by about 170% after treatment with CS in comparison with non-transformed roots. In fact, the three enzymes were associated with several stress-related processes including root browning, would healing, and disease resistance through the activation of plant defense responses [44]. CS elicited an increase in total flavonolignans in *Silybum marianum* HRs by switching on the lignification process involving the three key enzymes [45,46]. In summary, it is possible that the improvement in GIA was because PA plays a role as a source material for the production of numerous flavonoid and phenolic compounds through the reaction of phenylalanine ammonia lyase. Afterwards, CS feeding was conducted to trigger the defense response of AHRs to produce more specific secondary metabolites related to GIA. As shown in Table 3, the synergistic effect of PA and CTS had significant positive correlations with GIA, FLA, and PHEL. Moreover, the Pearson r value was higher than that achieved with individual CS feeding, and it achieved equal value to that seen in the growth kinetics experiment. In a previous study of precursor combined with elicitor feeding, a sufficient synergistic effect was confirmed when utilized at an appropriate dosage and time point. For example, by adding both an elicitor (acetylsalicylic acid) and precursors (PA + cysteine), the production of glucotropaeolin in *Tropaeolum majus* HRs increased by up to 4.8 times of that obtained when each factor was used alone [47]. In cell suspension cultures of *V. vinifera*, PA and methyl jasmonate had a remarkable synergistic effect, leading to an increase in anthocyanin production of 4.6 times that of the control [48].

## 4. Materials and Methods

### 4.1. Preparation of Ethanol Extract

The dried AHRs were ground into a fine powder using a blender. The coarse powder was extracted with absolute ethanol at a ratio of 1:10 (w/v) using the maceration technique [49]. Following that, the mixture was filtered, and the extract was collected using a vacuum rotary evaporator (HS-2005S-N, Hahnshin Scientific Company) at 50 °C. This process was repeated 3 times for all samples.

### 4.2. α-Glucosidase Inhibitory Activity of Ethanol Extract of Urena lobata

The α-glucosidase (EC 3.2.1.20) inhibitory activity (GIA) was assessed by the standard method [50] with slight modifications. The ethanol extract was dissolved in 5% (v/v) dimethyl sulfoxide at various concentrations. The α-glucosidase (Sigma, America) solution (1.0 U/mL) and 5 mM p-nitrophenyl-α-D-glucopyranoside (pNPG) (Sigma, America) solution were prepared in phosphate buffer (0.1 M and pH 6.8). A mixture containing 50 μL of the sample solution and 50 μL of the enzyme solution was incubated for 20 min, and then 40 μL of pNPG solution was added. The reaction occurred for 20 min and was then terminated by the addition of 130 μL of 0.2 M Na_2_CO_3_ (Sigma, America). Finally, the reaction mixture was measured at 405 nm using an ELISA Microplate Reader (DAS company, Italy). All steps of the enzyme experiment were implemented in the incubator (IN—IF Memmert Company) to maintain a temperature of 37 °C. Acarbose (Glucobay, India) was used as the standard. The inhibition of α-glucosidase (%) was calculated using the following formula:

(1) $$\%\;\mathrm{Inhibition}=\frac{\left(\mathrm{OD}\;\mathrm{control}-\mathrm{OD}\;\mathrm{blank}\;\mathrm{control}\right)-\left(\mathrm{OD}\;\mathrm{sample}-\mathrm{OD}\;\mathrm{blank}\;\mathrm{sample}\right)}{\;\left(\mathrm{OD}\;\mathrm{control}-\mathrm{OD}\;\mathrm{blank}\;\mathrm{control}\right)}\times 100\%$$

where the OD control and sample represent the absorbance of the control and sample, respectively, and OD blank control and blank sample represent the absorbance of the control and sample, respectively, without the addition of the enzyme solution (replaced by phosphate buffer). The concentration of inhibitors required for inhibiting 50% of the α-glucosidase activity under the assay conditions was defined as the IC_50_ value (µg/mL). For reasons of clarity, we used a value of 1/IC_50_ or GIA ((µg/mL)^−1^) for all data analysis; the larger the GIA, the more efficient the inhibition of enzyme.

### 4.3. Determination of the Phenolic Content

PHEL was determined by mixing the root extract with Folin–Ciocalteu reagent. After 5 min, 2% (w/v) Na_2_CO_3_ was added into the mixture which was then continuously incubated for 45 min at room temperature. The absorbance of the reaction mixture was measured at 765 nm, and gallic acid (GA) was used as the standard (0–50 µg/mL) [51].

### 4.4. Determination of the Flavonoid Content

FLA was quantified by using the aluminum chloride method [52] with several slight modifications. The root extract was mixed with 5% NaNO_2_ (w/v) for 5 min. Following that, 10% AlCl_3_ (w/v) and 1 M NaOH were added into the mixture. The absorbance of the reaction mixture was measured at 510 nm and quercetin (Q) was used as a standard (0–100 µg/mL).

### 4.5. Preparation of Agrobacterium rhizogenes

*Agrobacterium rhizogenes* ATCC 15834 was obtained from the RIKEN bank through the Japanese Government (MEXT) project. *Agrobacterium rhizogenes* was grown in liquid yeast mannitol medium (YMB) (Sigma, America) (yeast extract 10 g/L, mannitol 10 g/L, K_2_HPO_4_ 0.5 g/L, MgSO_4_.7H_2_O 0.2 g/L, NaCl 0.1 g/L, pH 7.0) [53] with shaking (110 rpm) at 25 ± 1 °C until the OD_600_ of bacterial suspension was 0.6.

### 4.6. Establishment of the Aeroponics System

**Figure 9:**
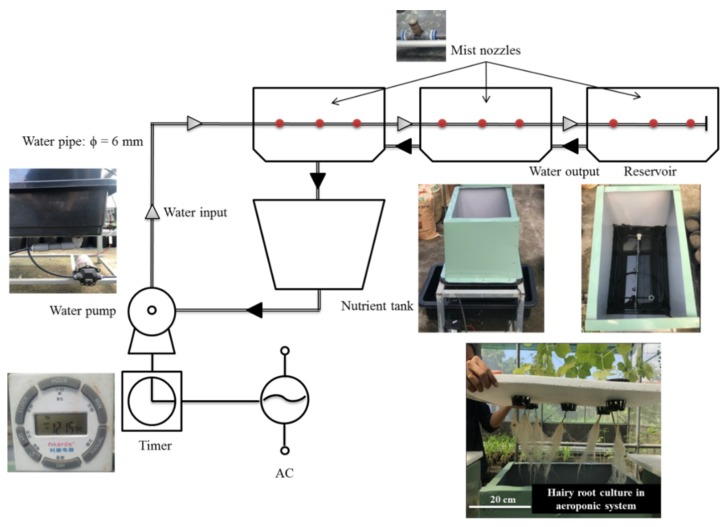
Aeroponics system. φ: diameter, AC: alternating current.

The aeroponics system (Figure 9) was designed by Plant Biotechnology Laboratory, University of Science—Vietnam National University. It contains 3 mist nozzles per reservoir with a spraying capacity located at the bottom of the reservoir (80 cm height × 40 cm width × 60 cm length). Nutrient solution was added into a tank (20 cm height × 40 cm width × 60 cm length) and then transferred to the reservoirs using a water pump (HT-75 24V 1.8 L/minutes, Taiwan). A timer (Kerde TC-932, China) was set up for spraying nutrient solution every 10 min for 30 s into the inner part of the reservoir in order to keep the roots wet.

### 4.7. Optimization of Exogenous Factors for the AHRs and rol Gene Detection

All culture experiments were conducted from 1 March to 30 September in the greenhouse. Seeds were germinated on a soil tray. The original procedure for aeroponic hairy root cultures of *Urena lobata* can be briefly described as follows: 15 day old plantlets were induced by *Agrobacterium rhizogenes* ATCC 15834 following our previously published procedure [13]. Specifically, the *Urena lobata* plantlets had their roots cut off, wounding the hypocotyls, which were dipped into the *Agrobacterium rhizogenes* ATCC15834 suspension (OD_600_ = 0.6). After an infection time of 10 min, the infected plantlets were inoculated for 5 days on MS medium. Then, the inoculated plantlets were washed three times with water containing 250 mg/L of cefotaxime to eliminate *Agrobacterium rhizogenes*. Finally, all plants were transferred into aeroponics systems containing a nutrient solution (pH 5.5–6.0), EC (Electrical Conductivity) = Ms cm^−l^, NO_3_^−^, SO_4_^2−^, H_2_PO_4_^−^, Cl^−^, K^+^, Ca^2+^, Mg^2+^, NH_4_^+^, Na^+^, and micronutrients with the concentrations equal to liquid MS medium. The aeroponic medium culture was diluted following a ratio of 0.8 L nutrients:19.2 L water (1/25). The rooting time (RT) was determined by observing the emergence of the first root from the infection sites during inoculation. The rooting rate was determined after 4 days of infection. The fresh weight (FW) and dry weight (DW) were measured after 30 days of being transferred to the aeroponics system.

To enhance the rooting efficiency and growth, different infection time periods (10 min, 30 min, and 60 min), media (MS, B5 and WPM) and ratios of medium salt strength (1/10, 1/25 and 1/50) were investigated. After optimizing each factor, the optimal factors were selected and combined to generate the optimized procedure that was compared with the original procedure.

The integration of T-DNA into genomic plant DNA was confirmed by PCR analysis using *rol*B, *rol*C, and *vir*D specific primers in accordance with our previous study [13]. Specific primers were used for amplifying *rol*B (5′-GCTCTTGCAGTGCTAGATTT-3′ and 5′-GAAGGTGCAAGCTACCTCTC-3′), *rol*C (5′-CTCCTGACATCAAACTCGTC-3′ and 5′-TGCTTCGAGTTATGGGTACA-3′), and *vir*G (5′-TTATCTGAGTGAAGTCGTCTCAGG-3′ and 5′-CGTCGCCTGAGATTAAGTGTC-3′) [13]. Genomic DNA from non-transformed roots and transformed roots was extracted using CTAB, as described by Edwards et al. [54]. For amplification, the PCR program included a step of 5 min at 94 °C and 35 cycles (each consisting of 1 min at 94 °C, 1 min at 55 °C, and 1 min at 72 °C), followed by a final extension at 72 °C for 10 min. PCR products were analyzed after electrophoresis in a 1% agarose gel.

### 4.8. Kinetics of Growth, α-Glucosidase Inhibitory Activity, Phenolics and Flavonoids Contents of Urena lobata Hairy Root

After transfer into an aeroponics system, all plant samples were cultivated with a plant density of 20 cm × 20 cm and put into a reservoir with a total of 10 plants per reservoir. The temperature was maintained between 25 °C and 28 °C by a sensor and cooling system (Model: DMV-DLE5065/11, EU). The plants grew under natural light conditions without additional light; 2–3 cm of each particle clay was used as a substrate. Fungicide and insecticide treatments were applied when appropriate. For each 2 weeks of culture, the biomass of AHRs was determined for 12 weeks.

### 4.9. Precursor and Elicitor Preparation and Application

To study the effect of the precursor treatment concentration, PA (Sigma, America) was dissolved with distilled water and then added into the medium on the first day of aeroponics culture to give final concentrations of 1 mM, 10 mM, 100 mM, and 200 mM. The AHRs of all treatments were obtained at week 10.

To study the effects of CTS and ET, CS (from shrimp shells, ≥75% deacetylated, Sigma, America) was dissolved in glacial acetic acid (1%, v/v) and then added into the medium on the 8th week of aeroponics culture to give final concentrations of 50 mM, 100 mM, and 150 mM. After 5 days, 10 days, 15 days, or 20 days of elicitation, the AHRs of all treatments were harvested.

### 4.10. Data Processing and Statistical Analysis

The AHRs in all treatments were evaluated in term of the DW (g), GIA ((µg/mL)^−1^), PHEL (mg GA g^−1^ DW), and FLA (mg Q g^−1^ DW) by one-way analysis of variance (ANOVA) using the SPSS 16.0 statistical software (©2007 SPSS Inc.). Values are the mean of three replicates from three experiments, with each replicate containing 30 samples. The data was processed statistically by analysis of variance (ANOVA) and difference between means of the samples analyzed by the least significant difference (LSD) at a probability level of 0.05.

## 5. Conclusions

We suggested an efficient procedure to increase the transformation rate of *Agrobacterium rhizogenes* to *Urena lobata* plantlets in green house conditions. The plantlets were soaked in bacterial suspension for 10 min and then cultured in WPM medium with a ratio of salt to water of 1:25. The established AHRs showed higher GIA than in vitro HRs and non-transformed roots. Therefore, we investigated whether the bioactivity value of AHRs could be increased by using precursor and elicitor feeding strategies. The GIA of AHRs was affected by the PA (precursor) and CS (elicitor). All the individually tested PA and CS treatments significantly increased the DW, GIA, FLA, and PHEL. Regarding the individual treatments, the strongest increases in DW, GIA, FLA and PHEL were detected from the AHRs with the addition of 10 mM PA, while 15 days of elicitation with 50 mM CS was the most effective treatment. Furthermore, the combination of PA (10 mM) with CS (50 mM + 15 days of elicitation) showed the best improvement, remarkably increasing all parameters compared with the individual treatments. To the best of our knowledge, the farming strategy of secondary metabolites through the combined use of PA and CS feeding is practical for *Urena lobata* AHRs culture.

## Figures and Tables

**Table 1:** Effects of the infection time, type of medium, and ratio of mineral salt strength on the efficient transformation and growth of *Urena lobata* aeroponic hairy roots (AHRs) after 30 days of culture.

	RT (Days)	RR after 4 Days (%)	FW after 30 Days (g)	DW after 30 Days (g)
**Infection Time (Minutes)**				
0	5.33 ± 0.58 a	0.00 ± 0.00 c	61.58 ± 4.03 d	2.32 ± 0.21 d
10	3.00 ± 0.00 b	100.00 ± 0.00 a	114.42 ± 3.57 a	4.93 ± 0.13 a
30	3.00 ± 0.00 b	100.00 ± 0.00 a	95.41 ± 2.85 b	4.29 ± 0.16 b
60	4.67 ± 0.58 a	53.33 ± 15.28 b	80.27 ± 1.93 c	3.15 ± 0.18 c
**Type of Medium**				
MS	3.33 ± 0.58 b	93.33 ± 5.77 a	68.40 ± 4.65 b	2.35 ± 0.22 a
B5	5.00 ± 0.00 a	73.33 ± 5.77 b	64.62 ± 4.02 b	1.20 ± 0.18 b
WPM	2.33 ± 0.58 c	100.00 ± 0.00 a	84.59 ± 3.27 a	2.60 ± 0.22 a
**Mineral Salt Strength: Water (v/v)**				
1/10	3.00 ± 0.00 a	73.33 ± 5.77 b	65.85 ± 2.39 b	2.22 ± 0.74 b
1/25	2.67 ± 0.58 a	100.00 ± 0.00 a	102.80 ± 2.69 a	5.18 ± 0.16 a
1/50	3.33 ± 0.58 a	93.33 ± 5.77 a	32.74 ± 2.26 c	1.20 ± 0.39 b

The values in a line marked with different lower-case letters denote significant differences between samples at *p* < 0.05 (Duncan’s multiple range test). B5: Gamborg B5; DW: dry weight; FW: fresh weight; MS: Murashige and Skoog; RR: rooting rate; RT: rooting time; WPM: Woody plant medium.

**Table 2:** Comparison of the efficient transformation and growth of *Urena lobata* AHRs between the original and optimized procedures.

	RT (Days)	RR after 4 Days (%)	FW after 30 Days (g)	DW after 30 Days (g)
Control (aeroponic non-transformed root)	5.33 ± 0.58 a	0.00 ± 0.00 b	61.58 ± 4.03 c	2.32 ± 0.21 b
AHRs in original procedure (30 min: MS: 1/25)	3.00 ± 0.00 b	100.00 ± 0.00 a	68.28 ± 2.24 b	2.43 ± 0.07 b
AHRs in optimized procedure (10 min: WPM: 1/25)	2.33 ± 0.58 b	100.00 ± 0.00 a	130.39 ± 1.83 a	7.24 ± 0.26 a

The values in a line marked with different lower-case letters denote significant differences between samples at *p* < 0.05 (Duncan’s multiple range test). B5: Gamborg B5; DW: dry weight; FW: fresh weight; MS: Murashige and Skoog; RR: rooting rate; RT: rooting time; WPM: Woody plant medium.

**Table 3:** Correlation analysis between the GIA, FLA and PHEL of AHRs with growth kinetics, various individual treatments, and the combined effect of phenylalanine and chitosan.

r	DW	GIA	FLA
**Growth Kinetics (n = 18 samples)**			
GIA	0.963 ^**^		
FLA	0.777 ^**^	0.848 ^**^	
PHEL	0.981 ^**^	0.956 ^**^	0.794 ^**^
**Treatment with Phenylalanine (n = 15 samples)**			
GIA	ns		
FLA	0.627 ^*^	ns	
PHEL	ns	ns	0.884 ^*^
**Treatment with Chitosan Dosages and Exposure Time (n = 48 samples)**			
GIA	ns		
FLA	ns	0.575 ^**^	
PHEL	ns	0.756 ^**^	0.706 ^**^
**Combined Effect (n = 12 samples)**			
GIA	ns		
FLA	0.755 ^**^	0.778 ^**^	
PHEL	ns	0.970 ^**^	0.771 ^**^

r: Pearson’s coefficient, DW: dry weight, GIA: α-glucosidase inhibitory activity (1/IC_50_), FLA: flavonoid content, PHEL: phenolic content, ns: non-significant value, ^*^: correlation is significant at the 0.05 level, ^**^: correlation is significant at the 0.01 level.

## Abbreviations

**AHRs**	aeroponic hairy roots
**ATCC**	American type culture collection
**B5**	Gamborg B5 medium
**CS**	chitosan
**CTAB**	cetyl trimethylammonium bromide
**CTS**	chitosan dosage
**DW**	dry weight
**ET**	exposure time
**FLA**	total flavonoids content
**FW**	fresh weight
**GA**	gallic acid
**GIA**	α-glucosidase inhibitory activity (1/IC_50_)
**HRs**	hairy roots
**IC_50_**	the half maximal inhibitory concentration
**MS**	Murashige and Skoog medium
**PA**	L-phenylalanine
**PCR**	polymerase chain reaction
**PHEL**	total phenolics content
**Q**	quercetin
**r**	Pearson’s coefficient
**RR**	rooting rate
**RT**	rooting time
**WPM**	Woody plant medium
